# New Generation Federated Learning

**DOI:** 10.3390/s22218475

**Published:** 2022-11-03

**Authors:** Boyuan Li, Shengbo Chen, Zihao Peng

**Affiliations:** 1 School of Computer and Information Engineering, Henan University, Kaifeng 475001, China; 2 School of Mathematics and Computer Science, Nanchang University, Nanchang 330031, China

**Keywords:** federated learning, incremental learning, federated class-incremental learning, scalable network

## Abstract

With the development of the Internet of things (IoT), federated learning (FL) has received increasing attention as a distributed machine learning (ML) framework that does not require data exchange. However, current FL frameworks follow an idealized setup in which the task size is fixed and the storage space is unlimited, which is impossible in the real world. In fact, new classes of these participating clients always emerge over time, and some samples are overwritten or discarded due to storage limitations. We urgently need a new framework to adapt to the dynamic task sequences and strict storage constraints in the real world. Continuous learning or incremental learning is the ultimate goal of deep learning, and we introduce incremental learning into FL to describe a new federated learning framework. New generation federated learning (NGFL) is probably the most desirable framework for FL, in which, in addition to the basic task of training the server, each client needs to learn its private tasks, which arrive continuously independent of communication with the server. We give a rigorous mathematical representation of this framework, detail several major challenges faced under this framework, and address the main challenges of combining incremental learning with federated learning (aggregation of heterogeneous output layers and the task transformation mutual knowledge problem), and show the lower and upper baselines of the framework.

## 1. Introduction

Federated learning (FL) has received extensive attention since it was first proposed [1]. It breaks the shackles of the original machine learning, does not require centralized training of data, and achieves the effect of protecting privacy and breaking data silos. The privacy protection provided by it provides a great guarantee for the participating clients of joint modeling. It has made good achievements in several fields, such as finance, medical care [2], unmanned driving, etc.

The current bottlenecks of FL include communication bottlenecks [3] and heterogeneity bottlenecks. The communication bottleneck generally refers to the problem of limited communication bandwidth when a large number of IoT devices participate. Two methods are usually adopted. One is to quantify the model to compress, and the other is to randomly select clients to reduce participation. Another bottleneck is the non-IID data because the distribution of data in different fields is usually different, and the heterogeneity of the data will lead to the drift of the aggregation model. In addition, there is another biggest limitation of FL at this stage—that is, the task scale is fixed. Typically, due to the limitations of the tasks presented by the server, the model may be limited to an initialized size that lacks generalization capabilities, and there is also an idealized setting for client-side storage, which is no upper limit for client-side image storage. In fact, real-world interactive clients are not subject to these constraints, and more of these clients will learn more tasks online. These participating clients come from different devices, and it is impossible to have the same computing power and storage capacity as high-performance servers. In the case of continuous learning, these terminals may discard some outdated data to ensure the novelty of the model. However, because the arrival of tasks is unknown, it may cause a sharp drop in performance on old tasks. Such dynamic task-driven clients account for the vast majority of the real world, and they provide distinctive private data. We prefer that they can participate in the FL process without the interference of intermediaries or agents.

**Figure 1:**
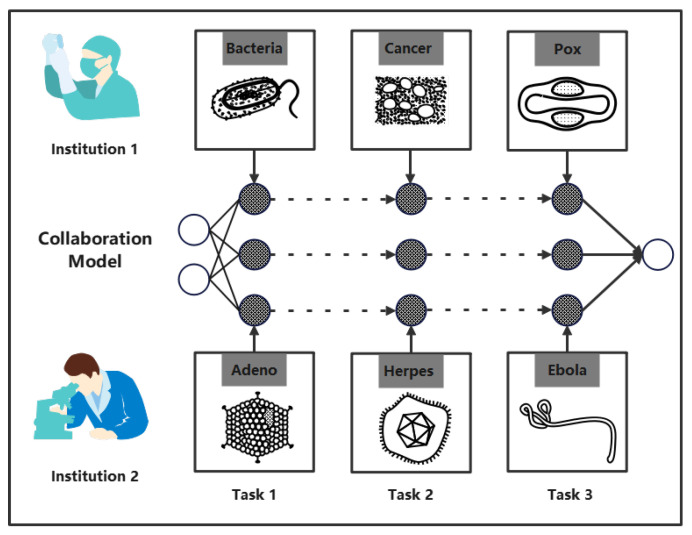
Joint modeling for different stages of disease research from different institutions.

To solve this problem, we propose a new federated learning paradigm, called the new generation federated learning (NGFL). NGFL will have an important reference value in future federated learning work. We introduce incremental learning into federated learning and describe a real-world scenario in which it might happen. In the NGFL, each participating terminal will no longer be constrained by the task of the server and can incorporate its own private tasks into the model, making the server-aggregated model more powerful. More specifically, these clients will dynamically participate in federated learning and receive tasks dynamically. The tasks received online by themselves are not independent and identically distributed (non-iid), as are the tasks received between each client. We consider the scenarios under the data collaboration of different hospitals in Figure 1. Among different specialized hospitals, data collaboration cannot be achieved due to different fields; the amount of medical data is huge, and one-time training is time consuming and labor intensive. For example, the rapid mutation of COVID-19 is a headache for many researchers. Some studies have linked neural networks to drug development and have achieved good results [4], but we found that the need for the amount of training data is huge, which may be as high as tens of trillions of bytes. As COVID-19 mutates, more and more features need to be added to the network and trained, and de novo training is almost impossible because it is time-consuming [5]. Due to the gap between research institutions, some key results may not be shared immediately. For another example, with the rapid development of the monkeypox virus, traditional machine learning cannot achieve rapid knowledge conversion. All reasons lead to the inefficiency of the existing federated learning.

Little research has been done on this work at the current stage, and there is no strict definition of this problem. A very intuitive method is to directly combine class-incremental learning and federated learning for cross-application. Only one recent study partially explains it [6], but it still doesn’t address some of the core issues, (i.e., it does not provide a solution for the fusion of heterogeneous output layers after incremental learning). We found that the existing class incremental learning has made good progress [7,8,9,10], but only solves the catastrophic forgetting of the local client. These problems will be exacerbated in federated learning, because each communication with the server needs to fuse the model, which will make such an over-parameterized model central to the calculation. In addition, some clients will be suspended from interacting due to limited communication, and these clients will continue to receive tasks before communicating again. The best way to deal with these continuously received tasks still needs careful consideration.

As a fundamental framework for the new generation federated learning, we address for the first time the core problems of incremental and federated learning, making it possible for applications. In order to enable better research in future work, we describe the various problems faced in detail in the text and give the performance lower and upper bounds of the algorithm under this framework.

The main contributions of our work are as follows:

- We combine incremental learning with federated learning and propose a new generation federated learning, which is dedicated to solving cross-client knowledge increments. It will have the possibility to expand the knowledge with the interaction of different clients.
- We summarize and define some of the most important challenges facing the NGFL framework and present our proposed basic solutions. Various baseline schemes are designed according to the solution to these challenges, and the optimal upper bound of the algorithm is given for research.
- For the first time, we address the problem of model aggregation at the incremental fusion stage.

This paper will be described in the following sections. Related works are surveyed in Section 2. Section 3 defines the problem. Section 4 presents and addresses the challenges respectively. Numerical experiments are presented in Section 5. Finally, Section 6 concludes and discusses the paper, and Section 7 points out some of the limitations of this paper and the outlook for the future.

## 2. Related Works

### 2.1. Class-Incremental Learning

Incremental learning refers to retaining the previously learned knowledge after learning new knowledge that comes continuously [11,12]. This learning method is closer to the human model. As a challenge of deep learning, it only accesses the data of the current task each time, which may bring catastrophic forgetting. Many studies address this challenge from multiple perspectives, such as [7,8,9,10,13,14,15,16,17,18,19,20,21]. Some research [11,22,23,24,25,26,27] uses saving or generating a small number of samples to prevent the network from forgetting previous tasks, and iCaRL [11] is the cornerstone of this approach, and has been used in many studies.

### 2.2. Federated Learning

FL [1] offers the possibility of joint modeling across data. Data heterogeneity as a key issue has attracted much attention, and many studies have attempted to address this issue [28,29,30,31,32,33,34]. FedProx [35], attempts to exploit the constraints of the two-norm to correct for local training objectives. Paper [36,37] uses a small amount of shared data and Bayesian estimation to revise the local model and the global model. In communication, ASO-fed [38] mitigates the straggler problem caused by device heterogeneity. Paper [39] proposed an asynchronous algorithm that keeps its convergence speed consistent with the synchronous algorithm in all client communication situations. DC-ASGD [40] proposes a novel technology to compensate delay caused by asynchronous learning.

Obviously, none of these works can be adapted to client-side streaming tasks.

### 2.3. Federated Class-Incremental Learning

To the best of our knowledge, there is little research in this area. Literature [6] is the only one that defines the problem of class incremental learning in federated learning. It proposed the method of global–local compensation for the forgetting problem, but there are some idealistic assumptions, and the definition is not comprehensive enough, overlooking some key issues.

## 3. Problem Definition

Table 1 describes some subscripts used in this paper.

### 3.1. Class-Incremental Learning

In the traditional single-node scenario for incremental learning, we denote the task stream as $T={\left\{T^{t}\right\}}_{t=1}^{T}$, where *T* denotes the number of tasks. For the *t*-th task $T^{t}$, it contains *K* data samples, which can be denoted as $T^{t}={\left\{x_{k}^{t},y_{k}^{t}\right\}}_{k=1}^{K}$, where $x_{k}^{t}\in X^{t},y_{k}^{t}\in Y^{t}$, and $X^{t},Y^{t}$ denote the sample space and label space of the *t*-th task respectively. Note that $Y^{t}$ may contain duplicate label elements, and we use $\left\{Y^{t}\right\}$ to denote the aggregated results. For example, assume $Y^{t}=\left[0,0,1,2,3,3\right]$, and then we have $\left\{Y^{t}\right\}=\left\{0,1,2,3\right\}$ and $|\{Y^{t}\}|=4$. We define the *t*th task to contain $|C^{t}|$ new classes, where $C^{t}={\left\{c_{a}^{t}\right\}}_{a=1}^{\left|\{Y^{t}\}\right|}$ is the set of classes of the *t*th task, and $c_{a}^{t}$ denotes the *a*-th class of the *t*th task $T^{t}$. Moreover, we define the set of all classes in the first *t* − 1 tasks as $C^{p}=\sum_{u=1}^{t-1}C^{u}\subset Y^{p}$, where $Y^{p}=\sum_{v=1}^{t-1}Y^{v}$ denotes the previous *t* − 1 tasks in the overall label space. We can express it in more detail as $C^{p}={\left\{c_{a}^{p}\right\}}_{a=1}^{\left|\{Y^{p}\}\right|}$, with $c_{a}^{p}$ denoted as the *a*th class in the previous *t* − 1 tasks. When a node performs task *t*, it can only access the current task $T^{t}$, and this task does not overlap with the previous task (i.e., $C^{t}\cap C^{p}=\emptyset$, $C^{t}\cup C^{p}=\left\{c_{1}^{p},..c_{\left|\{Y^{p}\}\right|}^{p},c_{|\{Y^{p}\}|+1}^{t},,,..c_{|\left\{Y^{p}\right\}\left|+\right|\left\{Y^{t}\right\}|}^{t}\right\}$), and we denote the total number of all task classes including task t as, $H^{t}=\sum_{1:t}|C^{t}\left|=\right|C^{t}\left|+\right|C^{p}|$.

### 3.2. Federated Learning

We describe a traditional FL process. We assume a total of N clients, and each client owns a private dataset $D_{i}={\left\{x_{k},y_{k}\right\}}_{k=1}^{K}$. We aimed to get the global optimal $\Theta^{*}$ over the global dataset $D=\cup_{i\in [N]}D_{i}$ contains |C| classes by

(1) $$\begin{array}{c} \Theta^{*}=\underset{\Theta }{\arg\min}\ell \left(\Theta \right)=\sum_{i=1}^{N}\frac{|D_{i}|}{|D|K}\sum_{k=1}^{K}\ell_{i}\left(o\left(x_{i,k};\Theta_{i}\right),y_{i,k}\right), \end{array}$$

where $\Theta_{i}$ is the machine learning model to be optimized and $\ell_{i}:R^{D}\to R$ is the local loss function on client *i*’s dataset $D_{i}$, $o\in R^{\left|C\right|}$, which denotes the probability predicted via Θ, and *ℓ* is the empirical loss on the global dataset D.

### 3.3. Model Decoupling

To better describe incremental learning, we decouple the model classification layer from the feature extraction layer. First, we define a machine learning model whose output is represented as $o\left(x\right)=o\left(x;\Theta \right)\in R^{\left|C\right|}$, where *x* is represented as the input data, Θ as the model parameters and $R^{\left|C\right|}$ as the output vector space. Furthermore, we decompose the model into two parts. The first part of the model is the feature extractor, which is represented as $\Phi \left(x\right)=\Phi \left(x;\Upsilon \right)=z:R^{D}\to R^{d}$, Υ denotes the parameters of the feature extractor, *z* is the input information transformed by the feature extraction, $R^{D}/R^{d}$ denotes the vector space before and after the transformation, respectively. The second part of the model is the classifier, which is represented as $\Gamma \left(z;\Omega \right):R^{d}\to R^{\left|C\right|}$ (Ω denotes the parameters of the classifier, |C| denotes the number of categories the classifier can carry, and the classifier input is the output of the feature extractor *z*). We can therefore rewrite this model as o(x)=o(x;Θ)=Γ(Φ(x;Υ),Ω). Finally, the label $\hat{y}$ is predicted by σ(o(x)) and σ denotes the softmax function. It should be noted that due to the nature of incremental learning, the output layer dimension varies as the task continues to arrive.

### 3.4. New Generation Federated Learning

**Figure 2:**
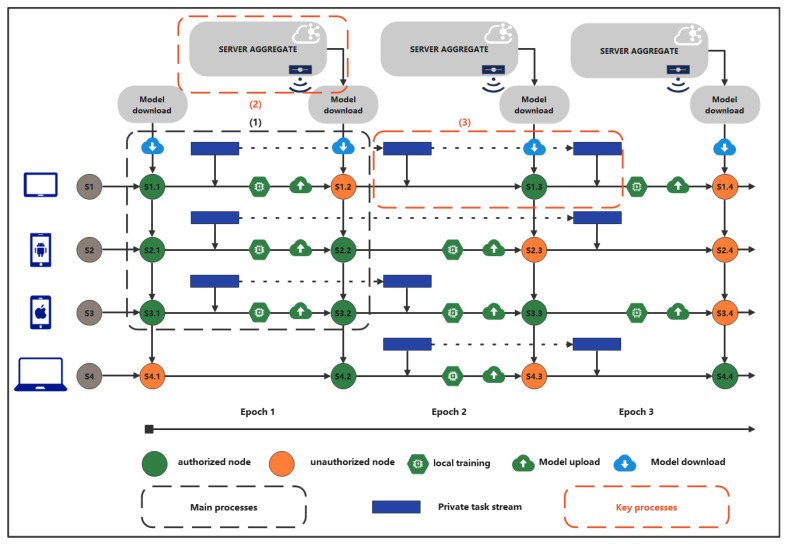
New generation federated learning workflow.

We illustrate the NGFL workflow in Figure 2, which contains a total of four randomly selected clients, and there are a total of three communication rounds. Unlike the current federated learning, this definition is more in line with the real world, which will have dynamic task flow and space storage constraints.

We extend incremental learning to a federated learning environment and characterize a new generation of federated learning. We first describe the dynamic participation of clients, which has N±N clients and is denoted as ${\left\{S_{i}\right\}}_{i=1}^{N\pm N}$, with ±N denoting the random withdrawal or addition of some clients as communication proceeds. We now formally describe its unique dynamic tasking feature, wherein a server $S_{G}$ exists to coordinate and communicate with the clients, with a total of r={1,…,R} rounds for global communication. For each client, $S_{i}$, it has a private task stream $T_{i}={\left\{T_{i}^{t}\right\}}_{t=1}^{T}$. Each client will receive the global model $\Theta^{r}$ from the server $S_{G}$ in the first *r* communication round, update it with its own task $T_{i}$ and obtain $\Theta_{i}^{r}$. Each client then transfers the local model $\Theta_{i}^{r}$ to the server for aggregation and downloads the model again.

In particular, the server is also an incremental learning process at the macro level. As it communicates with clients, it gains more knowledge from model aggregation and gradually expands the network. We denote the server-specific task flow as $T_{S_{G}}={\left\{T_{S_{G}}^{t}\right\}}_{t\propto \{r,T_{i}\}}^{T}$, with $T_{S_{G}}^{t}$ denoting the server’s *t*th task. Note that although we define a server-side task flow, this task flow is virtual and it increases as it interacts with the client. Specifically, $t\propto \{r,T_{i}\}$ means that its incremental tasks are coupled with communication rounds *r* and client-side tasks $T_{i}$. If no increment exists on the client side, then the server model will remain constant as communication proceeds. If there are client-side increments and no communication, then the server model does not change either. That is, $T_{S_{G}}^{r,t}=\sum T_{i}^{r,t}$, in the *r*th round in which the server incremental tasks come from the sum of all client incremental tasks that participated in the communication in this round, and vary with the communication. In contrast, the client’s task flow $T_{i}$ is completely independent and autonomous, and it is not influenced by any factor. Precisely because we do not have any a priori knowledge about client tasks, a client may face (0…n) tasks in the *r*th round, which we denote as

(2) $$\begin{array}{c} T_{i}^{r,\overset{˚}{t}}=\left\{\overset{each\;round\;r}{\overset{︷}{\emptyset ,T_{i}^{r,t},T_{i}^{r,t+1}+\cdots ,T_{i}^{r,t+n}}}\right\}. \end{array}$$

$T_{i}^{r,\overset{˚}{t}}$ denotes the set of all tasks encountered by client *i* in the *r*th round. Unlike the independent task ID *t*, we use $\overset{˚}{t}$ to denote the macro representation of all tasks in this communication. That is, each client task in round *r* satisfies $T_{i}^{r,t}\subset T_{i}^{r,\overset{˚}{t}}$, and the server tasks can be further written as $T_{S_{G}}^{r,t}=\sum_{i\in S}T_{i}^{r,\overset{˚}{t}}$ (the sum of all tasks of the authorised client *i* in the *r*th round).

Specifically, for client *i*’s *t*th task $T_{i}^{r,t}$ in round *r* of communication, its label space $Y_{i}^{r,t}\subset Y_{i}^{r,\overset{˚}{t}}$. Consistent with the single-node scenario, the client *i*’s *t*th task in the *r*-th round $T_{i}^{r,t}$ contains $|C_{i}^{r,t}|$ new classes and differs from its previous t−1 tasks $C_{i}^{p}=\sum_{u=1}^{t-1}C_{i}^{u}\subset \sum_{v=1}^{t-1}Y_{i}^{v}$. After loading the global model $\Theta^{r}$, each client $S_{i}$ performs the incremental task set $T_{i}^{\overset{˚}{t}}$ and obtains $\Theta_{i}^{r,\overset{˚}{t}}$. Then, all clients $\left\{S_{i}\right\}$ upload their $\Theta_{i}^{r,\overset{˚}{t}}$ to the server $S_{G}$ to aggregate into the global model $\Theta^{r+1}$. The server will distribute $\Theta^{r+1}$ to the client in the next round of communication. Note that, unlike individual nodes, we define the total number of categories for which client $S_{i}$ contains task t as $H_{i}^{t}=|C_{i}^{t}\left|+\right|C_{i}^{p}\left|+\right|C_{S_{G}}^{r,p}\left|-\right|\left(C_{i}^{t}\cup C_{i}^{p}\right)\cap C_{S_{G}}^{r,p}|$ ($C_{S_{G}}^{r,p}$ means class that the server $S_{G}$ learns from all clients in the previous r−1 rounds), i.e. as communication proceeds, the tasks viewed by client *i* come not only from itself, but also from the tasks of other clients involved in the communication; the reasons are given in Section 4.2.

**Figure 3:**
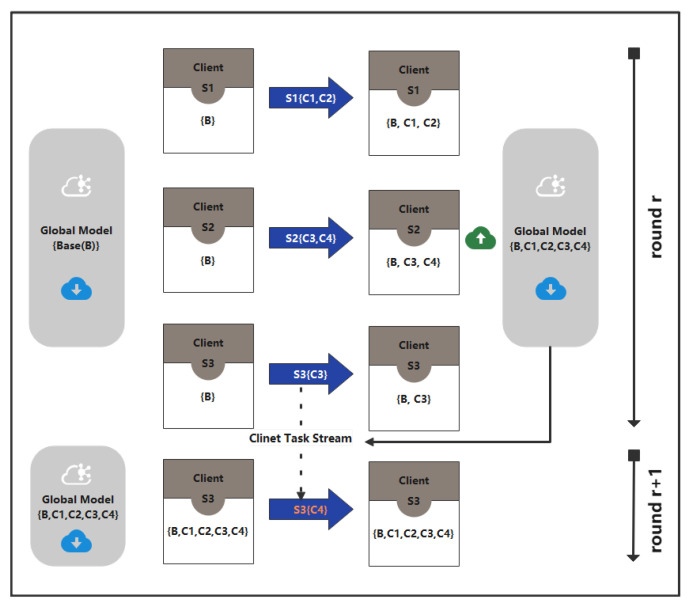
Description of the workflow.

For example, we will elaborate the "Main Process(1)" which in the upper part of Figure 3. Like traditional federated learning, the server will initialize the global model for delivery. This initialization model has the initial basic task (B) shown in the leftmost part of Figure 3. Because they are limited by the server’s field of view, more tasks will be learned from different clients $S_{i}$. These clients will receive the initialization task model $\Theta^{r}$ from the server and perform local updates. According to standard incremental learning, during each communication between the server and clients, the client may face a different private incremental task, (e.g., C3,C4, $S_{i}\left\{\cdot \right\}$ represents private data belonging to class C of client *i*), or there may be no additional classification task. Each client will form a model that is incrementally updated locally, and then upload it to the server. These models will be automatically merged by the server to generate a new global model $\Theta^{r+1}$.

So we have the objective mean loss function that needs to be optimized at any point time:

(3) $$\begin{array}{c} L_{t}=\frac{1}{\left|S\right|}\sum_{i\in S}\frac{1}{H_{i}^{t}}\sum_{j=1}^{H_{i}^{t}}\frac{1}{K}\sum_{k=1}^{K}\ell_{i}\left(o\left(x_{i,j,k}\right),y_{i,j,k}\right), \end{array}$$

where *i* represents the client and *j* represents the classes learned from the local and server.

The new generation of federated learning should achieve the following effects.

- The server only needs to be given a basic task. More tasks should be learned from participating clients.
- The server should aggregate the models of different private tasks in the client, and achieve good performance.
- Every client should not have catastrophic forgetting.
- Clients should have good knowledge of other tasks from the global model.
- This framework should apply to all algorithms in this field.

## 4. Important Issues

We will address some important issues facing the new generation federated learning.

### 4.1. Client & Server Forget

**Figure 4:**
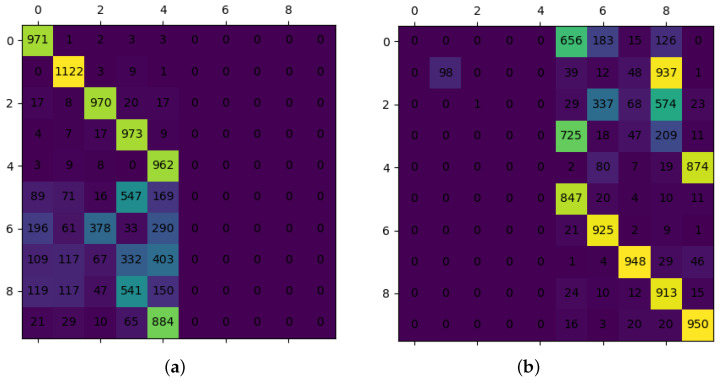
Simple incremental task flow in groups of five classes. (**a**) Incremental task1. (**b**) Incremental task2.

Catastrophic forgetting on the part of the client as tasks continue to arrive is a common problem in incremental learning. That is, in cases where the old and new tasks do not overlap, the learned knowledge decays rapidly if nothing is done about it. As shown in Figure 4, we look at a test confusion matrix with two incremental tasks and clearly see that the previous task will no longer have discriminability except for the latest task, which has a clear diagonal correct rate.

The same is true on the server side, where some knowledge will be forgotten due to the heterogeneity of the client-side data and aggregation. This forgetting will be exacerbated by the forgetfulness of dynamic tasks on the client’s side.

#### Solution: Self-Attention and Total Attention

To solve this problem, we propose a variant of the loss function. In many studies of incremental learning, an immediate idea is to use cross-entropy to compute the loss that takes into account all viewed classes, but this may bring attention bias. We will naturally think of two ways to calculate cross-entropy. The first is for all observed classes (total attention (TA)):

(4) $$\begin{array}{c} \ell_{i}\left(x,y;\Theta^{t}\right)=\sum_{h=1}^{H_{i}^{t}}y_{h}log\frac{exp(o_{h})}{\sum_{h^{\prime }=1}^{H_{i}^{t}}exp\left(o_{h^{\prime }}\right)}. \end{array}$$

Note that in this case, back-propagation of gradients will be done on all classes exposed, so these loss errors will be back-propagated from all outputs, including those that do not belong to the current task.

Differently from the first method, we only consider the current task, and change the cross-entropy calculation to the following form (self-attention (SA)):

(5) $$\begin{array}{c} {\tilde{\ell }}_{i}\left(x,y;\Theta^{t}\right)=\sum_{h=1}^{|C_{i}^{t}|}y_{\left[H^{t-1}+h\right]}log\frac{exp(o_{\left[H^{t-1}+h\right]})}{\sum_{h^{\prime }=1}^{|C_{i}^{t}|}exp\left(o_{\left[H^{t-1}+h^{\prime }\right]}\right)}. \end{array}$$

The premise of this variant application is that we do not consider the replay of any old tasks, and if some old tasks are added, Equation (4) must still be used.

### 4.2. Task Overlap

Due to the definition of incremental learning, with the progress of task stream and communication, the tasks of each client do not overlap. That is, each node needs to perform incremental operations on the classification layer for every task. However, compared to the global model, there may be other clients that have completed incremental for their own tasks’ class, so it is necessary to distinguish between true incremental tasks and pseudo-incremental tasks to avoid unnecessary overhead.

As shown in the lower part of Figure 3, if we assume that client S3 receives model $\Theta^{r}$ from the server in the *r*th round, it executes incremental task $T_{S3}^{t}=C3$ locally and obtains model $\Theta_{S3}^{r,t}=\left\{B,C3\right\}$. After the r+1 round of server fusion, model $\Theta^{r+1}=\left\{B,C1,C2,C3,C4\right\}$ is delivered again. At this point, client S3 executes the second incremental task $T_{S3}^{t+1}$= C4, which is completely new from the perspective of the client S3’s own task stream. However, from the perspective of the server, this tasks’ class has been incrementally completed by the client S2 in round *r*, as shown in Figure 3. Client S3 does not need to increment the network; it can be trained directly.

Therefore, there are three scenarios for client-side incremental tasks:

1. Full-covered: The entire class of client $S_{i}$ in the latest private task $T_{i}^{t}$ in round *r* has been incremented by other clients in the previous r−1 rounds, i.e. there are no new incremented classes. It can be expressed as
  (6) $$\begin{array}{c} C_{i}^{r,t}\subset C_{S_{G}}^{r,p}. \end{array}$$
2. Semi-covered: Some of the classes in client $S_{i}$’s latest private task $T_{i}^{t}$ in round *r* have been incremented by other clients in the previous r−1 rounds, i.e. there are some new incremented classes. It can be expressed as
  (7) $$\begin{array}{c} C_{i}^{r,t}\cap C_{S_{G}}^{r,p}\neq \emptyset . \end{array}$$
3. Not covered: The latest private task $T_{i}^{t}$ of client $S_{i}$ in round *r* has not been incremented by other clients in the previous r−1 rounds, i.e. all are new incremental classes. It can be expressed as
  (8) $$\begin{array}{c} C_{i}^{r,t}\cap C_{S_{G}}^{r,p}=\emptyset . \end{array}$$

#### 4.2.1. Solution: Double-Ended Task Table

In order to solve the problem of task intercommunication between the client and the server, we propose a task alignment table. Similar to the routing table used to record port numbers and destination addresses, this task alignment table exists on both the server side and the client side. On the client side, this table records all task classes covered up to the current task and the corresponding neural network classifier for each class. At the end of local training, the client uploads this table along with the model to the server, which maintains a global alignment table based on the task table submitted by the client and aligns the network output layers. The server then fuses the model based on this table and sends the table down with the model at the same time, and the client updates the local alignment table for the next round of training. This task alignment table is indispensable as the basis for cross-application of federation learning and incremental learning, and lays the foundation for the model fusion that follows.

### 4.3. Model Aggregate

As shown in "Key Process (2)" in Figure 2, because the task flow is invisible and the arrival time is unknown for each client—that is, the classifier output dimensions are different after updating the client’s local task—it is possible that the same dimensions represent different outputs. Specifically, as shown in Section 3.3, the aggregation of models in traditional federation learning satisfies

(9) $$\begin{array}{c} \Theta^{r+1}=\frac{1}{N}\sum_{i\in S}\Theta_{i}^{r}=\frac{1}{N}\sum_{i\in S}\left(\Upsilon_{i}^{r}⊕\Omega_{i}^{r}\right). \end{array}$$

Normally, the model does not change in federation learning, and a simple aggregation is all that is required. However, due to the nature of incremental learning, different clients have feature extractors Φ(·;Υ) with the same dimensionality, but have classifiers Γ(z;Ω) with different structures. Therefore, we need a strategy to aggregate this heterogeneous structure, and in the next subsections we will elaborate two strategies to solve this problem.

#### 4.3.1. Solution: Pre-Alignment and Post-Alignment

**Figure 5:**
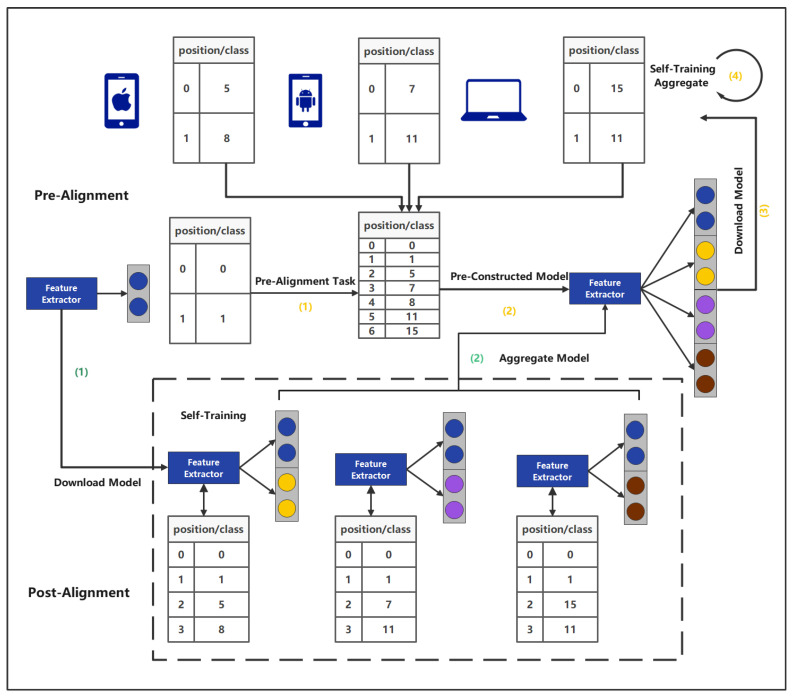
Pre-alignment and post-alignment workflow.

In the existing research, to the best of our knowledge there is no work to illustrate it. We elaborate on the two alignment works at the network levels because in incremental learning, the output layer network gradually increases as new tasks arrive. Clients will have their own special output layer structure, and because each client is independent of the other, they will have completely different output layers. Most studies ignore this issue, and they assume that the server is done incrementally to the output layer when the model is delivered, which is unreasonable. We elaborate on this; first, we define a task alignment table in Section 4.2.1 to allow each client to articulate its own output meaning. Its work flow is shown in Figure 5.

##### Pre-Alignment (PreA)

We use the PreA method, aiming for the server to confirm the client has seen all incremental tasks before each time the server authorizes communication, which to pre-define the output layer when the model is broadcast. The advantage of this method is that the average strategy can be used directly when the network is aggregated. However, this also brings a big problem, that is, the predefined incremental network cannot be increased with the arrival of tasks. Tasks gradually come in the once communication is temporarily held or discarded until the next communication.

##### Post-Alignment (PostA)

The second method is the PostA method. When the server communicates, the model is sent to the client with the task alignment table. The client has enough freedom to arbitrarily change the output layer until the model is uploaded with its task table during communication. At this time, the server needs to adjust and aggregate the output layer according to the alignment table submitted by the client. The advantage of this is that the client can perform incremental learning completely autonomously and can perform asynchronous operations, but the server requires more cost and may perform at a lower level.

#### 4.3.2. Solution: Partial Fusion and Total Fusion

For this heterogeneous output layer, we choose two aggregation strategies. We express it with the following equation:

(10) $$\begin{array}{c} \Theta^{r+1}=\frac{1}{\left|S\right|}\sum_{i\in S}\Theta_{i}^{r}⊙M_{i}=\frac{1}{\left|S\right|}\sum_{i\in S}\left(\Upsilon_{i}^{r}⊕\left(\Omega_{i}^{r}⊙M_{i}\right)\right). \end{array}$$

$M_{i}$ represents the fusion mask of client *i*’s current task (Partial-Fusion **PF**) or all viewed classes (Total-Fusion **TF**). Specifically, $M_{i}={𝟙}_{\left[C_{S_{G}}^{r}\in C_{i}^{r,\overset{˚}{t}}\right]}\in R^{d\times |C_{S_{G}}^{r}|}$, where ${𝟙}_{\left[\cdot \right]}$ is an evaluation function used to test whether the subscript factor satisfies the condition, ${𝟙}_{\left[True\right]}=1$; otherwise, ${𝟙}_{\left[False\right]}=0$. The fusion mask is constructed by a double-ended task table and does not require the client to submit additional information.

### 4.4. Communication & Storage Limit

Limited by the communication bottleneck, it is impossible for the server to select all clients and then select a subset of them at random. Those clients which are not selected may come to some key private tasks and have uniqueness, so this random selection leads to fatal omissions. The client itself may not know the importance of these data and discard it limited by storage space.

Specifically, the server will select a random subset of clients $S_{r}$ for each communication round *r*, and those unselected clients are recorded as $S_{r}^{c}$ (complement of $S_{r}$). We assume a client $i\in S_{r}^{c}$ missed authorization at the *r*th round, but get the authorization at the $r{}^{\prime }$th round. As shown in "Key Process (3)" in Figure 2, because we don’t have any prior knowledge about the task stream, we don’t know if the client will overwrite or drop some tasks due to memory constraints as the stream of tasks keeps arriving in the communication from round *r* to $r^{\prime }$.

We do not examine this, as this paper centers on how to integrate incremental learning into FL.

## 5. Experiments

For NGFL, we adopt a set of lower bound baselines, and a set of upper bound baselines to demonstrate its performance. For the upper baseline, to allow for clear target boundaries for subsequent studies we give two reasonable upper bounds for the framework to be investigated. Some of the work takes the visible results of all tasks as an unacceptable upper bound, and we do not require that such a framework can immediately achieve the performance results of integrated learning. First, we argue that all clients can effectively and unrestrictedly use their own data from all old tasks as a first upper bound, Upper-Baseline(Self), which effectively judges the effectiveness of the client’s algorithms in the face of incremental learning. The second is that all clients can use global participation in modeling other clients’ old task data, Upper-Baseline(Global), which is an effective way to judge the merits of model aggregation algorithms in the face of multi-client heterogeneity in incremental tasks.

For the lower baseline, we use the traditional federated learning method, that is, we use SGD locally and then perform average aggregation on the server. This baseline usually performs badly, so we also give a set of options in Section 4.1. This includes basic solutions to improve client-side forgetting and server-aggregation. We do not want to directly introduce the existing incremental learning method into the NGFL, because of the lack of interaction between clients, these basic solutions provide the direction for the NGFL research. Specifically, by using a variant of the loss function (Self-Attention(SA)) over the traditional baseline and an improved scheme (Partial-Fusion(PF)) of aggregate mode.

### 5.1. Datasets

We used two commonly used real-world datasets.

#### 5.1.1. CIFAR100

The CIFAR100 dataset has 100 classes. Each class has 600 color images of size 32 × 32, of which 500 are used as the training set and 100 are used as the testing set. For each sample, it has two labels, "fine_labels and coarse_labels", which represent the fine-grained and coarse-grained labels of the sample, respectively.

#### 5.1.2. Tiny-ImageNet

The Tiny-ImageNet dataset has 200 classes. Each class has 600 color images of size 64 × 64, of which 500 are used as the training set, 50 are used as the testing set and 50 for the validation set.

### 5.2. (Hyper)Parameters

In the experiment, there are some important parameters. R is the number of global communication rounds. E and BS denote the number of epochs and batch size for local training, respectively. LR is the learning rate, M is the momentum, W is the weight decay value, and the optimizer is set to the SGD optimizer.

For the CIFAR100 dataset, we set the total number of tasks to be 10. In the initial task, the global model will learn the knowledge of the initial 10 classes. The remaining other classes will be evenly distributed to P (P=(10−1)) in the incremental task pool, and each incremental task pool has PC (PC=(100−10)/(10−1)) classes. For the Tiny-ImageNet dataset, we set the total number of tasks to be 10. In the initial task, the global model will learn the knowledge of 20 classes. The remaining other classes will be evenly distributed to P (P=(10−1)). In the incremental task pool, each incremental task pool has PC (PC=(200−20)/(10−1)) classes.

We simulate the incremental tasks as follows. Each client randomly selects 60% of the incremental tasks from the incremental task pool as the client’s local incremental tasks. The random selection of incremental tasks ensures the objectivity of incremental task selection, and 60% is used as a larger incremental ratio to meet the possibility of many sudden increments in the objective world.

In the initial task, we set R = 50, E = 5, BS = 128, LR = 0.01, M = 0.9, and W = 5 $\times 10^{-4}$. In incremental tasks, we set E = 5, BS = 128, LR = 0.005, M = 0.9, W = 2 $\times 10^{-4}$, and set R = 10 (intensive task switching) and R=20 (sparse task switching) ) in two cases to conduct the experiment. The settings of CIFAR-100 and Tiny-ImageNet are consistent.

### 5.3. Baseline Analysis

**Figure 6:**
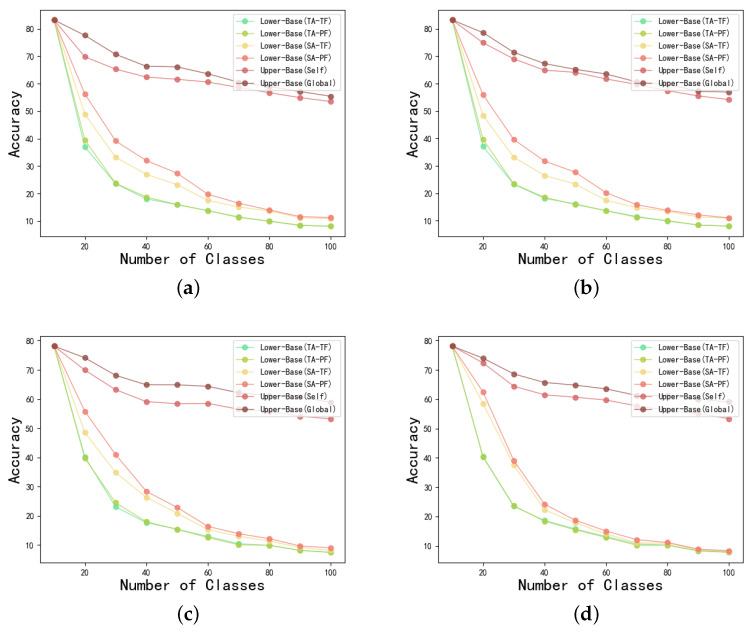
Experimental performance under different combinations. (**a**) CIFAR100: intensive task. (**b**) CIFAR100: sparse task. (**c**) Tiny-ImageNet: intensive task. (**d**) Tiny-ImageNet: sparse task.

Full experimental results are given in Table 2, Table 3, Table 4 and Table 5 and Figure 6.

For the low-base (TA-TF) setting, we found that in the absence of any constraints, experimental performance decreases sharply as the task sequence progresses. For example, in the intensive task assignment under the Cifar100 dataset in Table 2, the aggregation accuracy was as high as 83.20% for the first task but plummeted to 36.95% after the introduction of the second task sequence, and the model was only 8.04% accurate after the last task was completed. The most important reason for this phenomenon is that incremental tasks cannot overlap with previous tasks, and client-side incremental knowledge is also heterogeneous. To address this issue, we used a variant of the loss function introduced in Section 4.1 and found significantly better results in the first two task transformations. For example, in Table 4, the accuracy was 36.95% under the second task using the traditional loss function (TA-TF) and 48.94% with the variant of the loss function (TA-PF), with an 8% increase in accuracy under the third task and an average accuracy increase of about 5% over the full task. With the introduction of a partially fused server (TF / PF) strategy, we found a slight improvement in performance, e.g. an average increase in accuracy of about 1% in the full task in CIFAR100 (TA-TF/PF) and about 2% in the full task in (SA-TF/PF). We find that the effect is not significant because the main effect of this method is the fusion of heterogeneous outputs, a trend that is also satisfied with the Tiny-ImageNet dataset in Table 4 and Table 5.

For other upper-bound baseline settings, we find that the global-upper bound is slightly better than the self-upper bound, which is expected as it uses historical samples from all clients. For example, in Table 2, Table 3, Table 4 and Table 5, the accuracy improvement is about 2–3% in cifar100 and about 4–5% in Tiny-imgenet for both intensive and sparse tasks.

In addition, after changing the sparse task to the intensive task setting, we found that the upper baseline performance decreased, but the lower baseline performance did not change significantly. Moreover, in the partial lower baseline intensive task setting, there are also slightly better results than in the sparse task setting. For example, in the sparse task assignment in Table 3, after the first task is trained, the model has the same accuracy as the intensive task in Table 2, and after the second task is introduced the model has a 37.10% accuracy, which is a slight increase compared to the dense task, but as the task increases the accuracy decreases and the final accuracy is only 7.95%. This occurs because sparse task transitions give each task more time to train, but cause greater forgetting of the previous task.

## 6. Conclusions & Discussion

In this paper, we present a framework for the new generation federated learning that breaks the shackles of existing federated learning and enables free task integration. Unlike existing cross-applications of incremental and federated learning, which mainly address the problem of task forgetfulness while neglecting the primary problem of model heterogeneity, NGFL is a true combination that properly addresses the problem of task mutual knowledge and heterogeneous output fusion through task alignment tables.

We discuss a classic work as well as a recent one and describe their limitations. For example, one of the most popular research works is [11], which proposed a basic method of saving partial sample replay and achieved good results by combining it with knowledge distillation; many works were based on it. For the first time, it uses the feature extractor to obtain the average features of participating samples’ classes {C} by $\mu^{C}=\frac{1}{K}\sum_{x\in X^{C}}\Phi \left(x\right)$, and sorts them according to the two-norm (‖·‖) obtained by each type of sample, so as to select the best representative sample set for a single client. However, in federated learning, because of the diversity of participating clients, we prefer more features to participate in training, and this simple strategy lacking interaction ignores the distribution of global features and may lose some performance. The latest GLFC [6] progress also uses the example retention strategy of IcaRL and uses the same old model distillation knowledge supplement to complete the training strategy as ICaRL. In particular, the old model is the best fusion of the old global model in history ("Global Catastrophic Forgetting Compensation"). But the server requires definition of additional networks for the client to provide local example gradients and reconstruct example images. In terms of performance, this strategy has brought considerable benefits, but it still does not solve the fundamental problem of federated incremental learning (integration of heterogeneous models, knowledge interaction), and increases the overhead. None of them, including the latest work, properly describe and address the challenges we present, and we make it possible to combine federal learning with incremental learning.

## 7. Limitation & Future Works

As an exploration of the new generation of federation learning, this paper still has some limitations. For example, although we have addressed the basic problem of combining incremental learning with federated learning (aggregation of heterogeneous output layers), we have not fully addressed the challenge of knowledge forgetting and have only suggested possible research directions to address this challenge. In future work, we will propose theoretical support for the integration of incremental and federated learning, and conduct more research in, for example, incremental task interaction and heterogeneous knowledge fusion.

## Figures and Tables

**Table 1:** Some Subscript Descriptions.

Notation	Description
r=[1,…,R]	Global communication round
t=[1,…,T]	Client task id
e=[1,…,E]	Client local training round
$Y^{t}/C^{t}$	Label /class * space at *t*-th task
$Y_{i}^{t}/C_{i}^{t}$	Label/class space for client *i* at *t*-th task
$Y_{i}^{r,t}/C_{i}^{r,t}$	Label/class space for client *i* at *t*-th task in *r*-th round
$Y_{i}^{r,\overset{˚}{t}}/C_{i}^{r,\overset{˚}{t}}$	Label/class space for client *i*’s all task in *r*-th round

* Label space carries distribution information, whereas class space is purer. (i.e., $Y^{t}$ = [0, 0, 1, 2, 3, 3], $C^{t}$ = {0, 1, 2, 3}). All subscripts follow this naming rule.

**Table 2:** CIFAR100: Comprehensive performance on intensive tasks.

Methods	10	20	30	40	50	60	70	80	90	100	Avg.
Lower-Base(TA-TF)	83.20	36.95	23.56	18.02	15.91	13.71	11.42	9.86	8.32	8.04	22.89
Lower-Base(TA-PF)	83.20	39.50	23.70	18.67	15.97	13.71	11.29	9.93	8.42	8.04	23.24
Lower-Base(SA-TF)	83.20	48.94	33.19	26.97	23.18	17.48	15.08	13.61	11.13	10.84	28.37
Lower-Base(SA-PF)	83.20	56.30	39.13	31.79	27.39	19.71	16.45	13.95	11.58	11.2	31.06
Upper-Base(Self)	83.20	69.80	65.30	62.40	61.60	60.6	58.60	56.70	54.90	53.5	62.60
Upper-Base(Global)	83.20	77.60	70.70	66.32	66.11	63.59	60.54	58.95	57.11	55.41	65.90

**Table 3:** CIFAR100: Comprehensive performance on sparse tasks.

Methods	10	20	30	40	50	60	70	80	90	100	Avg.
Lower-Base(TA-TF)	83.20	37.10	23.29	18.15	16.07	13.65	11.51	9.84	8.39	7.95	22.91
Lower-Base(TA-PF)	83.20	39.70	23.40	18.50	15.90	13.60	11.25	10.0	8.38	8.00	23.19
Lower-Base(SA-TF)	83.20	48.40	33.16	26.49	23.34	17.45	14.72	13.41	11.35	10.88	28.24
Lower-Base(SA-PF)	83.20	55.94	39.63	31.72	27.68	20.20	15.80	13.77	12.11	11.00	31.08
Upper-Base(Self)	83.20	74.90	69.00	64.90	64.10	61.70	59.70	57.40	55.5	54.15	64.45
Upper-Base(Global)	83.20	78.60	71.40	67.30	65.20	63.50	60.60	59.80	57.20	56.90	66.37

**Table 4:** Tiny-ImageNet: Comprehensive performance on intensive tasks.

Methods	10	20	30	40	50	60	70	80	90	100	Avg.
Lower-Base(TA-TF)	78.10	40.20	23.10	17.67	15.45	12.95	10.44	9.91	8.23	7.49	22.35
Lower-Base(TA-PF)	78.10	39.80	24.50	18.00	15.37	12.58	10.10	9.91	8.16	7.50	22.40
Lower-Base(SA-TF)	78.10	48.50	34.90	26.30	20.90	15.30	13.00	11.40	9.16	8.17	26.57
Lower-Base(SA-PF)	78.10	55.80	41.00	28.40	22.90	16.40	13.90	12.17	9.71	9.03	28.74
Upper-Base(Self)	78.10	69.90	63.20	59.10	58.40	58.50	56.50	55.80	54.10	53.20	60.68
Upper-Base(Global)	78.10	74.10	68.10	64.87	64.85	64.36	62.11	61.16	60.65	58.85	65.71

**Table 5:** Tiny-ImageNet: Comprehensive performance on sparse tasks.

Methods	10	20	30	40	50	60	70	80	90	100	Avg.
Lower-Base (TA-TF)	78.10	40.30	23.49	18.60	15.70	13.15	10.59	10.23	8.28	7.85	22.63
Lower-Base (TA-PF)	78.10	40.50	23.63	18.37	15.45	12.83	10.18	10.17	8.23	7.73	22.52
Lower-Base (SA-TF)	78.10	58.60	37.50	22.27	17.82	14.04	11.00	10.86	8.61	8.04	26.68
Lower-Base (SA-PF)	78.10	62.50	39.06	24.20	18.70	15.03	12.08	11.11	8.85	8.26	27.78
Upper-Base (Self)	78.10	72.40	64.43	61.50	60.68	59.70	57.71	56.25	55.13	53.35	61.92
Upper-Base (Global)	78.10	74.00	68.63	65.70	64.80	63.51	61.28	61.22	60.05	59.03	65.63

## References

[1] McMahan B., Moore E., Ramage D., Hampson S., y Arcas B.A. Communication-Efficient Learning of Deep Networks from Decentralized Data. Proceedings of the 20th International Conference on Artificial Intelligence and Statistics (AISTATS).

[2] Guo P., Wang P., Zhou J., Jiang S., Patel V.M. Multi-Institutional Collaborations for Improving Deep Learning-Based Magnetic Resonance Image Reconstruction Using Federated Learning. Proceedings of the IEEE Conference on Computer Vision and Pattern Recognition, CVPR 2021.

[3] Paragliola G., Coronato A. (2022). Definition of a novel federated learning approach to reduce communication costs. Expert Syst. Appl..

[4] Lim J., Hwang S., Kim S., Moon S., Kim W.Y. (2019). Scaffold-based molecular design using graph generative model. arXiv.

[5] Bai X., Wang H., Ma L., Xu Y., Gan J., Fan Z., Yang F., Ma K., Yang J., Bai S. (2021). Advancing COVID-19 diagnosis with privacy-preserving collaboration in artificial intelligence. Nat. Mach. Intell..

[6] Dong J., Wang L., Fang Z., Sun G., Xu S., Wang X., Zhu Q. (2022). Federated Class-Incremental Learning. arXiv.

[7] Kirkpatrick J., Pascanu R., Rabinowitz N.C., Veness J., Desjardins G., Rusu A.A., Milan K., Quan J., Ramalho T., Grabska-Barwinska A. (2016). Overcoming catastrophic forgetting in neural networks. arXiv.

[8] Chaudhry A., Dokania P.K., Ajanthan T., Torr P.H.S. (2018). Riemannian Walk for Incremental Learning: Understanding Forgetting and Intransigence. arXiv.

[9] Lange M.D., Aljundi R., Masana M., Parisot S., Jia X., Leonardis A., Slabaugh G.G., Tuytelaars T. (2022). A Continual Learning Survey: Defying Forgetting in Classification Tasks. IEEE Trans. Pattern Anal. Mach. Intell..

[10] Aljundi R., Babiloni F., Elhoseiny M., Rohrbach M., Tuytelaars T. Memory Aware Synapses: Learning What (not) to Forget. Proceedings of the Computer Vision, ECCV 2018, 15th European Conference.

[11] Rebuffi S., Kolesnikov A., Sperl G., Lampert C.H. iCaRL: Incremental Classifier and Representation Learning. Proceedings of the 2017 IEEE Conference on Computer Vision and Pattern Recognition, CVPR 2017.

[12] Thrun S., Touretzky D.S., Mozer M., Hasselmo M.E. (1995). Is Learning The n-th Thing Any Easier Than Learning The First?. Proceedings of the Advances in Neural Information Processing Systems 8, NIPS.

[13] Zenke F., Poole B., Ganguli S. Continual Learning Through Synaptic Intelligence. Proceedings of the 34th International Conference on Machine Learning, ICML 2017.

[14] Jung H., Ju J., Jung M., Kim J. (2016). Less-forgetting Learning in Deep Neural Networks. arXiv.

[15] Li Z., Hoiem D. Learning Without Forgetting. Proceedings of the Computer Vision, ECCV 2016, 14th European Conference.

[16] Lee S., Kim J., Jun J., Ha J., Zhang B. Overcoming Catastrophic Forgetting by Incremental Moment Matching. Proceedings of the Advances in Neural Information Processing Systems 30: Annual Conference on Neural Information Processing Systems 2017.

[17] Liu X., Masana M., Herranz L., van de Weijer J., López A.M., Bagdanov A.D. Rotate your Networks: Better Weight Consolidation and Less Catastrophic Forgetting. Proceedings of the 24th International Conference on Pattern Recognition, ICPR 2018.

[18] Triki A.R., Aljundi R., Blaschko M.B., Tuytelaars T. Encoder Based Lifelong Learning. Proceedings of the IEEE International Conference on Computer Vision, ICCV 2017.

[19] Silver D.L., Mercer R.E. The Task Rehearsal Method of Life-Long Learning: Overcoming Impoverished Data. Proceedings of the Advances in Artificial Intelligence, 15th Conference of the Canadian Society for Computational Studies of Intelligence, AI 2002.

[20] Zhang J., Zhang J., Ghosh S., Li D., Tasci S., Heck L.P., Zhang H., Kuo C.J. (2019). Class-incremental Learning via Deep Model Consolidation. arXiv.

[21] Lee K., Lee K., Shin J., Lee H. Overcoming Catastrophic Forgetting With Unlabeled Data in the Wild. Proceedings of the 2019 IEEE/CVF International Conference on Computer Vision, ICCV 2019.

[22] Wu Y., Chen Y., Wang L., Ye Y., Liu Z., Guo Y., Fu Y. Large Scale Incremental Learning. Proceedings of the IEEE Conference on Computer Vision and Pattern Recognition, CVPR 2019.

[23] Castro F.M., Marín-Jiménez M.J., Guil N., Schmid C., Alahari K. End-to-End Incremental Learning. Proceedings of the Computer Vision, ECCV 2018, 15th European Conference.

[24] Shin H., Lee J.K., Kim J., Kim J. Continual Learning with Deep Generative Replay. Proceedings of the Advances in Neural Information Processing Systems 30: Annual Conference on Neural Information Processing Systems 2017.

[25] Ostapenko O., Puscas M.M., Klein T., Jähnichen P., Nabi M. (2019). Learning to Remember: A Synaptic Plasticity Driven Framework for Continual Learning. arXiv.

[26] Kemker R., Kanan C. (2017). FearNet: Brain-Inspired Model for Incremental Learning. arXiv.

[27] Xiang Y., Fu Y., Ji P., Huang H. Incremental Learning Using Conditional Adversarial Networks. Proceedings of the 2019 IEEE/CVF International Conference on Computer Vision, ICCV 2019.

[28] Kairouz P., McMahan H.B., Avent B., Bellet A., Bennis M., Bhagoji A.N., Bonawitz K., Charles Z., Cormode G., Cummings R. (2019). Advances and open problems in federated learning. arXiv.

[29] Li Q., Wen Z., Wu Z., Hu S., Wang N., Li Y., Liu X., He B. (2021). A survey on federated learning systems: Vision, hype and reality for data privacy and protection. IEEE Trans. Knowl. Data Eng..

[30] Li T., Sahu A.K., Talwalkar A., Smith V. (2020). Federated learning: Challenges, methods, and future directions. IEEE Signal Process. Mag..

[31] Yang Q., Liu Y., Chen T., Tong Y. (2019). Federated machine learning: Concept and applications. ACM Trans. Intell. Syst. Technol..

[32] Alazab M., RM S.P., Parimala M., Reddy P., Gadekallu T.R., Pham Q.V. (2022). Federated learning for cybersecurity: Concepts, challenges and future directions. IEEE Trans. Ind. Inform..

[33] Li A., Sun J., Li P., Pu Y., Li H., Chen Y. Hermes: An efficient federated learning framework for heterogeneous mobile clients. Proceedings of the 27th Annual International Conference on Mobile Computing and Networking.

[34] Li B., Chen S., Yu K. (2022). Model Fusion from Unauthorized Clients in Federated Learning. Mathematics.

[35] Sahu A.K., Li T., Sanjabi M., Zaheer M., Talwalkar A., Smith V. (2018). On the Convergence of Federated Optimization in Heterogeneous Networks. arXiv.

[36] Zhao Y., Li M., Lai L., Suda N., Civin D., Chandra V. (2018). Federated learning with non-iid data. arXiv.

[37] Chen H.Y., Chao W.L. (2020). Fedbe: Making bayesian model ensemble applicable to federated learning. arXiv.

[38] Chen Y., Nin Y., Slawski M., Rangwala H. (2020). Asynchronous Online Federated Learning for Edge Devices. arXiv.

[39] Avdiukhin D., Kasiviswanathan S., Meila M., Zhang T. (2021). Federated Learning under Arbitrary Communication Patterns. Proceedings of Machine Learning Research.

[40] Zheng S., Meng Q., Wang T., Chen W., Yu N., Ma Z.M., Liu T.Y. (2017). Asynchronous Stochastic Gradient Descent with Delay Compensation. Proceedings of Machine Learning Research.

